# Dietary iron attenuates epigenetic aging through DNA methylation remodeling and extends survival in older adults

**DOI:** 10.1186/s13148-025-01986-x

**Published:** 2025-10-29

**Authors:** Jia-Jun Zhao, Jianghua Zhang, Siyan Li, Qianqian Wang, Qiufen Mo, Huilin Yu

**Affiliations:** 1 College of Food and Health, Zhejiang A&F University, Hangzhou, Zhejiang China; 2 College of Food Science and Engineering, Central South University of Forest and Technology, Changsha, Hunan China; 3 College of Information Science and Technology, Donghua University, Shanghai, China

**Keywords:** Iron, DNA methylation, Epigenetic aging, Mortality, Nutrition

## Abstract

**Background:**

Iron homeostasis is essential for fundamental biological processes, yet its impact on epigenetic aging and mortality remains poorly understood. This study aimed to investigate associations between dietary iron intake and DNA methylation (DNAm) aging markers and to determine whether these epigenetic signatures mediate iron’s effects on mortality outcomes.

**Methods:**

We analyzed data from 2,398 adults aged ≥ 50 years in the National Health and Nutrition Examination Survey (1999–2002) with up to 20 years of mortality follow-up. Dietary iron intake was assessed through 24-h recall interviews. DNA methylation was profiled using the Illumina Infinium MethylationEPIC BeadChip. We employed multiple linear regression to identify iron-responsive DNAm features, Cox proportional hazards models to assess mortality associations, and formal mediation analyses to quantify epigenetic pathways.

**Results:**

Higher dietary iron intake was significantly associated with favorable epigenetic profiles, particularly with reduced levels of mortality-predictive DNAm markers GrimAge2Mort (*β* = −0.079, FDR = 0.00022), CRPMort (*β *= −0.072, FDR = 0.0037), and B2MMort (*β *= −0.057, FDR = 0.042). These iron-responsive DNAm features strongly predicted all-cause mortality (HRs per SD increase: 1.34, 1.21, and 1.08, respectively; all *p* < 0.05) and cause-specific mortality. Higher iron intake was directly associated with reduced risk of all-cause mortality (HR = 0.93 per SD increase, 95% CI 0.87–0.99), heart disease mortality (HR = 0.87, 95% CI 0.78–0.97), and respiratory disease mortality (HR = 0.72, 95% CI 0.56–0.93). Mediation analyses revealed that GrimAge2Mort mediated 22.7% of iron’s protective effect on all-cause mortality and 14.7% on heart disease mortality.

**Conclusions:**

This study establishes dietary iron as a modifiable determinant of epigenetic aging and mortality risk in older adults, with epigenetic recalibration mediating a substantial proportion of iron’s protective effects. These findings suggest optimizing iron intake may represent a promising nutritional strategy to promote healthy aging.

**Supplementary Information:**

The online version contains supplementary material available at 10.1186/s13148-025-01986-x.

## Introduction

Aging constitutes an irreversible biological process that serves as the strongest risk multiplier for chronic diseases, physical/cognitive decline, and mortality [1, 2]. Within three decades, 30% of the global population (2.1 billion) will be elderly—a transition positioning age-related morbidity as the defining public health challenge of our century [3]. While chronological age advances uniformly, biological aging rates diverge substantially across individuals due to modifiable factors including lifestyle and environmental exposures [4]. This variability underscores the critical need for robust biomarkers of biological aging [5]. Epigenetic clocks—particularly DNA methylation (DNAm)-based algorithms—have emerged as premier tools to quantify aging acceleration and predict mortality risk, outperforming conventional clinical biomarkers [6–8].

Iron homeostasis maintains fundamental biological processes through dual roles: as an enzymatic cofactor in DNA synthesis, energy metabolism, and oxygen transport and as a potential catalyst of oxidative damage [9, 10]. Preclinical evidence reveals paradoxical effects—dietary iron restriction extends lifespan in Caenorhabditis elegans, whereas iron repletion rescues mitochondrial dysfunction in aging rodents [11–13]. Human observational studies further complicate this picture: Both iron deficiency and overload correlate with increased mortality [14, 15]. These conflicting data underscore an unresolved question: Does iron exert bidirectional effects on aging trajectories through mechanisms beyond nutrient deficiency/toxicity?

Emerging evidence positions nutrition as a key modulator of epigenetic aging [16]. Mechanistically, iron may influence DNAm through two pathways: as a cofactor for ten-eleven translocation (TET) enzymes governing active demethylation, and via Fenton reaction-generated oxidative stress that disrupts methylation patterns [17–19]. Despite this biological plausibility, population studies specifically linking iron intake to epigenetic aging signatures remain absent. Crucially, the iron-epigenetic-mortality axis represents a fundamental knowledge gap with direct implications for dietary guidelines and aging interventions.

Here, we leverage the National Health and Nutrition Examination Survey (NHANES) cohort with linked mortality data to address three unmet challenges: (1) characterize dose–response relationships between dietary iron and established DNAm aging clocks; (2) validate the mortality-predictive capacity of iron-associated epigenetic signatures; (3) quantify the proportion of iron-mortality associations mediated through epigenetic aging pathways. Our findings establish iron as a modifiable determinant of epigenetic aging while providing the first evidence-based framework to optimize iron intake for healthy aging—delivering both biomarkers and intervention targets crucial for population-level geroprotection.

## Methods

### Study population and data source

We analyzed data from the 1999–2002 National Health and Nutrition Examination Survey (NHANES) cycles, which used a multistage probability sampling design to recruit 21,004 non-institutionalized U.S. adults [20]. All NHANES procedures were approved by the National Center for Health Statistics Research Ethics Review Board with documented informed consent (Protocols #98–12).

### Assessment of dietary iron intake and covariates

Dietary information was collected using 24-h dietary recall interviews conducted by trained interviewers using the USDA’s Automated Multiple-Pass Method [21]. Total daily iron intake (mean ± SD: 14.02 ± 8.43 mg/day) was calculated based on reported food consumption and supplement use. The raw distribution of iron intake is shown in Figure S1A. For analytical purposes, iron intake values were log-transformed, standardized using z-scores (standardized distribution shown in Figure S1B), and categorized into quartiles (Q1: < 8.39 mg; Q2: 8.39–12.03 mg; Q3: 12.03–17.34 mg; Q4: ≥ 17.35 mg) for subsequent analyses.

Baseline serum iron concentrations were measured through the DcX800 timed-endpoint assay (Beckman Coulter). Briefly, iron was dissociated from transferrin using acetic acid, reduced to Fe^2^⁺ with hydroxylamine/thioglycolate, and complexed with FerroZine reagent. Absorbance changes at 560 nm were quantified against calibration standards. Serum iron values were then categorized into quartiles (Q1: < 59 μg/dL; Q2: 59–81 μg/dL; Q3: 82–107 μg/dL; Q4: ≥ 108 μg/dL) for covariate adjustment.

Demographic and health-related information was obtained through structured interviews and physical examinations. Covariates included in our analyses were gender (male/female), race/ethnicity (non-Hispanic White, non-Hispanic Black, others), educational level (< high school, high school graduate, > high school), marriage status (married/living with partner, widowed/divorced/separated, never married), poverty-income ratio (PIR, categorized as < 1.3, 1.3–3.49, ≥ 3.5, which is an indicator of income relative to the economic needs of a household [22].), drinking status (never, former, current), smoking status (never, former, current), body mass index (BMI, < 25 kg/m^2^, 25–29.9 kg/m^2^, ≥ 30 kg/m^2^), and multimorbidity (categorized as 0, 1, 2, 3, ≥ 4 chronic conditions). Multimorbidity was assessed based on self-reported physician diagnoses of hypertension, heart disease, stroke, diabetes, cancer, chronic kidney disease, chronic obstructive pulmonary disease, and arthritis.

### DNA methylation profiling and epigenetic age markers

The study utilized DNA samples collected from adults aged ≥ 50 years in the NHANES 1999–2002 survey, employing a stratified sampling approach that included randomly selected non-Hispanic White participants (approximately 50% of eligible) and full enrollment of eligible participants from other racial/ethnic groups (non-Hispanic Black, Mexican American, other Hispanic, and other races). Whole blood samples underwent standardized DNA extraction protocols with subsequent cryopreservation at −80 °C until analysis.

DNA methylation (DNAm) profiling was conducted at Duke University’s Epigenetics Laboratory using the Illumina Infinium MethylationEPIC BeadChip v1.0 platform. Following manufacturer specifications, 500 ng of genomic DNA underwent bisulfite conversion (Zymo EZ Methylation kit) and PCR amplification (16 cycles: 95 °C/30 s, 50 °C/60 min). Converted DNA was processed through Illumina’s Infinium HD Methylation workflow, including overnight denaturation/amplification (20–24 h), enzymatic fragmentation, precipitation, and hybridization on EPIC BeadChips (16–24 h). Methylation data acquisition was performed using the Illumina iScan system with standard quality control metrics. Detailed information regarding the epigenetic data processing, including quality control, normalization, and adjustment for technical and biological covariates (e.g., cell type proportions, array position, plate, etc.), is comprehensively documented and available on the NHANES DNA Methylation Data Processing Website (https://wwwn.cdc.gov/nchs/nhanes/dnam/). These standard procedures applied by NHANES are designed to minimize batch effects and ensure data robustness.

Bioinformatic processing incorporated reference-based cell type deconvolution using the FlowSorted.Blood.EPIC_ref dataset and IDOL probe subset, implemented through the immunomethylomics package’s estimateCellCounts2 function [23–25]. Thirty established epigenetic biomarkers were derived through advanced regression modeling, integrating chronological age, sex, and CpG-specific methylation patterns. These included: HorvathAge, HannumAge, SkinBloodAge, PhenoAge, GDF15Mort, B2MMort, CystatinCMort, TIMP1Mort, ADMMort, PAI1Mort, LeptinMort, PACKYRSMort, CRPMort, logA1CMort, GrimAgeMort, GrimAge2Mort, HorvathTelo, YangCell, ZhangAge, LinAge, WeidnerAge, VidalBraloAge, DunedinPoAm, CD8TPP, CD4TPP, Nkcell, Bcell, MonoPP, and NeuPP. Cellular composition estimates and mortality-associated biomarkers (designated "Mort" suffix) were calibrated using regression algorithms accounting for technical and biological covariates. Details of each epigenetic biomarker are described in Table S1.

To minimize the influence of chronological age, each epigenetic marker was regressed on age, and the residuals were extracted and transformed to z-scores. The distributions of these DNAm biomarkers are shown in Figure S2.

To address multicollinearity among epigenetic markers and select a set of independent yet biologically meaningful features, we calculated a feature correlation distance matrix (1-r^2^) and performed hierarchical clustering using a 70% dissimilarity threshold. This approach allowed us to group highly correlated epigenetic markers and select the central feature from each cluster as a representative.

### Mortality assessment

Mortality status and cause of death were determined by linking NHANES participants to the National Death Index (NDI) through December 31, 2019, providing approximately 20 years of follow-up [26]. The primary outcome was all-cause mortality. Secondary outcomes included cause-specific mortality categorized according to the International Classification of Diseases, 10th Revision (ICD-10): heart diseases (I00-I09, I11, I13, I20-I51), malignant neoplasms (C00-C97), chronic lower respiratory diseases (J40-J47), cerebrovascular diseases (I60-I69), Alzheimer’s disease (G30), diabetes mellitus (E10-E14), influenza and pneumonia (J09-J18), nephritis/nephrotic syndrome/nephrosis (N00-N07, N17-N19, N25-N27), and other causes.

### Statistical Analysis

All statistical analyses were performed using R version 4.1.1. The FDR was calculated using the Benjamini–Hochberg procedure. The potential confounders included gender, race, educational level, marriage status, PIR, drinking status, smoking status, BMI, multimorbidity, serum iron levels as mentioned above.

To examine relationships between iron intake and DNA methylation features, we first visualized group differences using principal component analysis (PCA) and tested overall methylation profile differences across iron intake quartiles using permutational multivariate analysis of variance (PERMANOVA). We then employed Welch’s t-test to identify individual DNAm features that differed significantly between the lowest (Q1) and highest (Q4) iron intake quartiles, after ensuring approximate normality of the transformed features.

To quantify associations between iron intake and DNAm features while adjusting for potential confounders, we constructed multiple linear regression models with iron intake (continuous z-score) as the predictor and each DNAm feature as the outcome, adjusting for all covariates. We also explored potential nonlinear relationships using generalized additive models (GAMs). The significance of nonlinearity in GAMs was assessed using a likelihood ratio test comparing the GAM to a linear model, with the effective degrees of freedom (EDF) indicating the complexity of the fitted smooth term (EDF close to 1 suggests linearity, larger values indicate nonlinearity).

Cox proportional hazards models were used to examine associations between DNAm features and mortality outcomes, as well as between iron intake and mortality outcomes. All models were adjusted for the aforementioned covariates, and hazard ratios (HRs) with 95% confidence intervals (CIs) were reported. The proportional hazards assumption was verified using Schoenfeld residuals. Dose–response relationships between iron intake and mortality were further explored using restricted cubic spline models with three knots placed at the 25th, 50th, and 75th percentiles of dietary iron intake, which is a common practice to capture potential nonlinear associations across the data distribution.

To evaluate the predictive performance of DNAm features for mortality risk, we developed Survival Random Forest models. The dataset was randomly split into a ​training set (70%)​​ for model development and a ​test set (30%)​​ for independent validation. Discrimination was assessed within the test set using time-dependent area under the receiver operating characteristic (AUC) curves, calculated for 10-year survival. Predictive accuracy was quantified within the test set using Harrell’s C-index.

Finally, to investigate potential mediating roles of DNAm features in the relationship between iron intake and mortality outcomes, we conducted formal mediation analyses using the CMAverse package in R (version 0.1.0), which employs a counterfactual approach and allows for time-to-event outcomes. The average causal mediation effect (ACME), average direct effect (ADE), total effect, and proportion mediated were estimated with 95% confidence intervals based on 1,000 bootstrap samples, accounting for potential exposure-mediator interactions.

To assess whether baseline serum iron levels or cardiovascular disease status modified the observed relationships, we conducted interaction analyses by adding interaction terms to the aforementioned models.

All statistical tests were two-sided, and an FDR < 0.05 or *p*-value < 0.05 was considered statistically significant.

## Results

### Study population characteristics

**Fig. 1:**
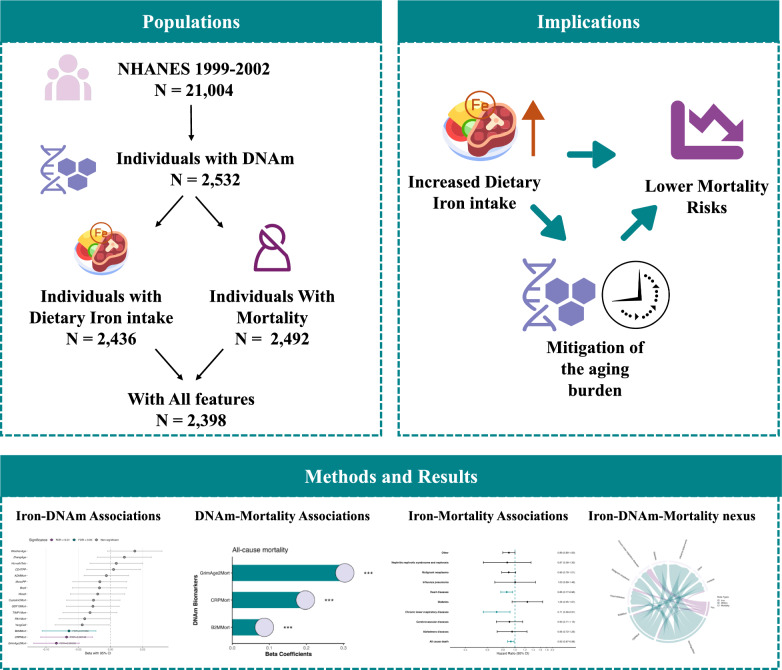
Study design and analytical framework. *(Left)* Participant selection flowchart, showing eligibility assessment from the NHANES cohort through methylation profiling to final analytical sample (*n *= 2,398). *(Bottom)* Schematic representation of analytical workflow examining associations between dietary iron intake, DNA methylation features, and mortality outcomes. *(Right)* Conceptual model illustrating the proposed pathway whereby increased dietary iron intake is associated with favorable DNA methylation profiles and reduced mortality risk

The baseline characteristics of the study population are summarized in Table S2. The participant selection flowchart and study design are detailed in ​Fig. 1. From the original NHANES cohort (*n* = 21,004), 2,532 individuals had valid DNAm profiles. Within this subsample: 2,436 provided complete dietary iron intake data, 2,492 had mortality follow-up records, and 2,398 met all mandatory inclusion criteria. The mean age of the final analytic sample was 66.13 ± 10.07 years, with 49.2% being female. The mean dietary iron intake from 24-h dietary recall interviews was 14.02 ± 8.43 mg.

Seventeen DNAm features were selected after dimensional reduction through clustering of features with > 70% dissimilarity based on correlation distance matrices (1-r^2^). The selected DNAm features showed low inter-correlation (all Spearman’s rho < 0.7, Figure S3), confirming their independence as epigenetic markers.

During the follow-up period (median 206 months), 1,321 deaths (53.0%) occurred. The most common cause of death was heart disease (27.1%), followed by malignant neoplasms, chronic lower respiratory diseases, and cerebrovascular diseases (Fig. 3a).

### Dietary iron intake is associated with favorable DNA methylation profiles

**Fig. 2:**
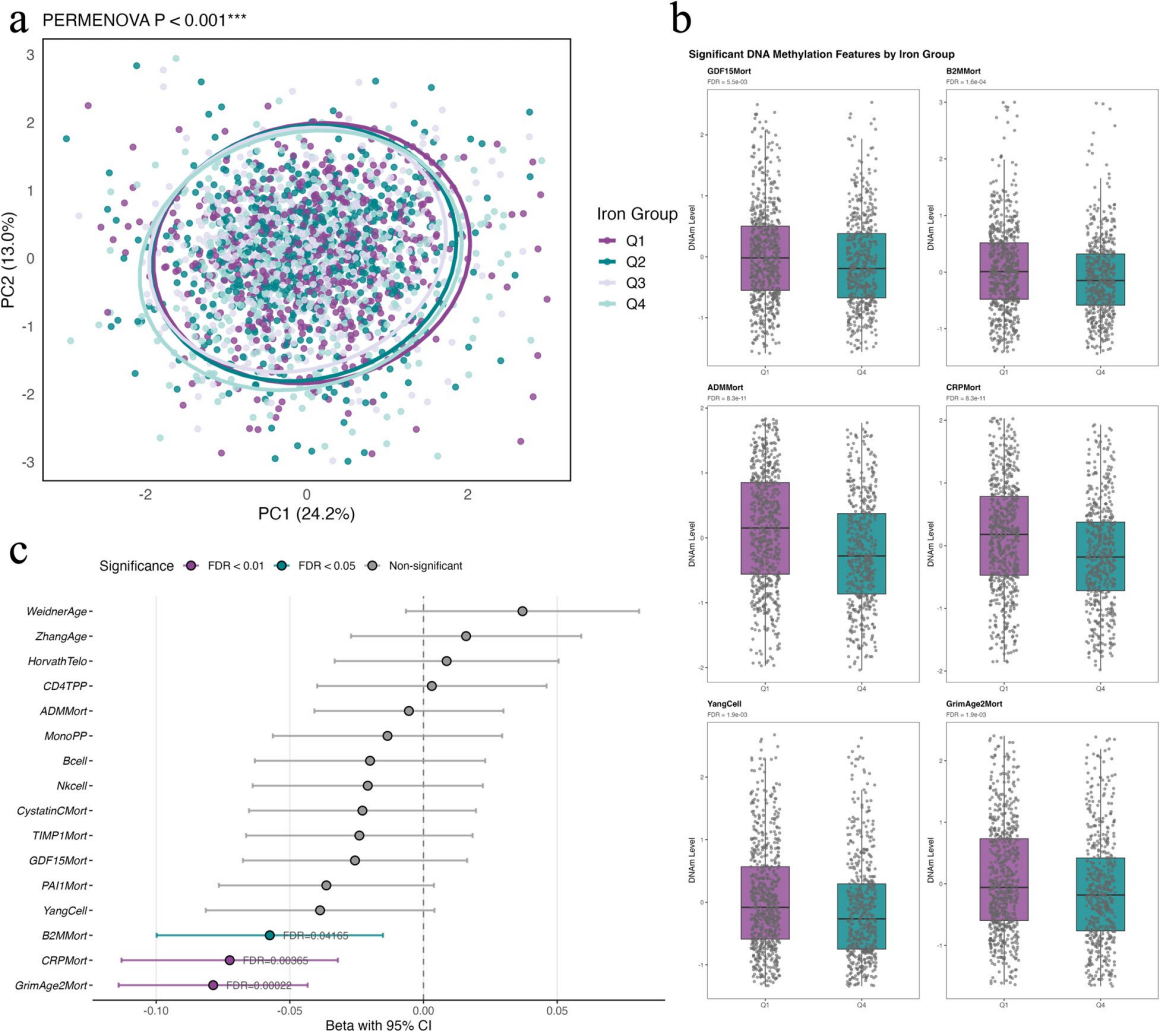
Associations between dietary iron intake and DNA methylation profiles. **a,** DNA methylation signature associated with dietary iron intake. a, Principal component analysis of 17 DNA methylation features showing significant separation between iron intake quartiles (PERMANOVA, Adonis, permutations = 999). **b,** Distribution of eight methylation features that significantly differed between lowest (Q1) and highest (Q4) iron intake quartiles (Wilcoxon rank-sum test, FDR < 0.05). **c,** Forest plot showing adjusted associations between dietary iron intake (standardized z-score) and DNA methylation features. Negative coefficients indicate inverse associations between iron intake and mortality-related DNA methylation markers. Models adjusted for gender, race, educational level, marriage status, PIR, drinking status, smoking status, BMI, multimorbidity, and baseline serum iron levels. Colors represent significance thresholds: purple (FDR < 0.01), green (FDR < 0.05), and gray (nonsignificant)

To investigate the relationship between iron intake and DNA methylation patterns, we first conducted principal component analysis (PCA) of the 17 DNAm features across iron intake quartiles. The PERMANOVA test revealed significant separation between iron intake groups (*p* = 0.001), indicating that iron intake explains a significant portion of the variance in DNAm profiles (Fig. 2a).

Further analysis using Welch’s t-test identified eight DNAm features that significantly differed across iron intake quartiles (FDR < 0.05) between Q1 and Q4. These included GDF15Mort, B2MMort, CystatinCMort, ADMMort, CRPMort, YangCell, Bcell, and GrimAge2Mort (Fig. 2b, Table S3). Notably, all of these markers showed decreasing trends with increasing iron intake, suggesting that higher iron intake is associated with more favorable epigenetic aging profiles.

To quantify these relationships while adjusting for potential confounders (gender, race, educational level, marriage status, poverty-income ratio, drinking status, smoking status, BMI, and multimorbidity), we performed multiple linear regression analysis. Three DNAm features remained significantly associated with iron intake after adjustment: GrimAge2Mort (*β *= −0.079, FDR = 0.00022), CRPMort (*β* = −0.072, FDR = 0.0037), and B2MMort (*β* = −0.057, FDR = 0.042) (Fig. 2c, Table S4). The negative coefficients indicate that increased iron intake is associated with decreased levels of these mortality-related DNAm markers.

We further explored potential nonlinear relationships using generalized additive models (GAMs). The results confirmed significant associations for GrimAge2Mort, CRPMort, and B2MMort (all FDR < 0.05). While GrimAge2Mort and CRPMort showed predominantly linear relationships with iron intake (EDF < 2), B2MMort exhibited a slightly more complex pattern (EDF = 2.67), with a modest upturn at very high iron levels that did not negate the overall inverse relationship (Figure S4).

### Iron-associated DNA methylation features predict mortality

**Fig. 3:**
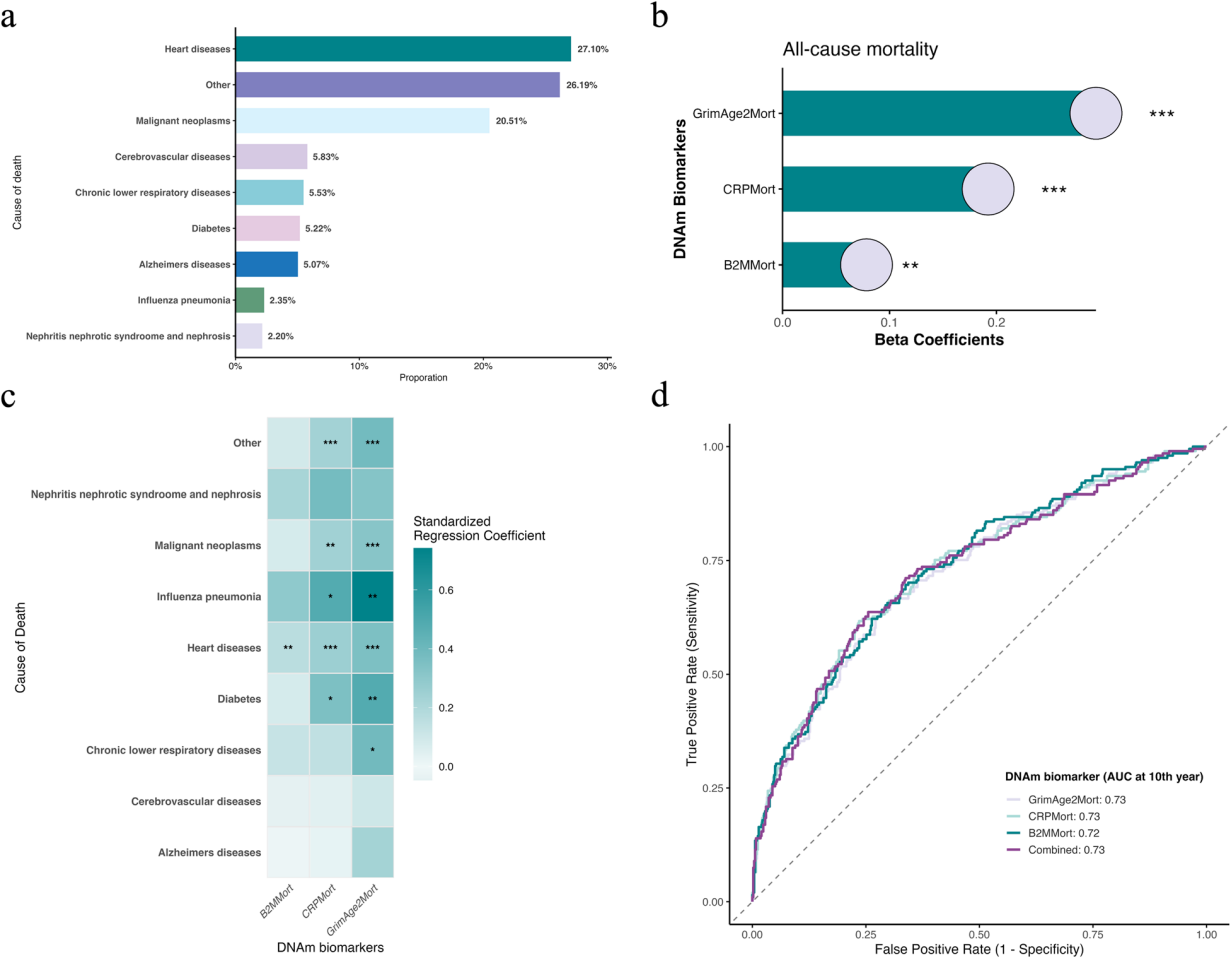
Mortality prediction by iron-responsive DNA methylation features. **a,** Distribution of cause-specific mortality among participants during follow-up (median 206 months), with heart diseases representing the leading cause of death (27.1%). **b,** Associations between iron-responsive DNA methylation features and all-cause mortality risk, presented as beta coefficients from Cox proportional hazards models adjusted for potential confounders. **c,** Heatmap showing standardized regression coefficients from adjusted Cox models for associations between iron-responsive DNA methylation features and cause-specific mortality. **d,** Receiver operating characteristic curves demonstrating predictive performance of iron-responsive DNA methylation features for all-cause mortality, with area under the curve (AUC) values indicated. *FDR < 0.05, **FDR < 0.01, ***FDR < 0.001

Having established associations between iron intake and specific DNAm features, we next investigated whether these iron-responsive DNAm markers could predict mortality outcomes. Cox proportional hazards models, adjusted for all covariates, revealed that GrimAge2Mort (*β* = 0.29, HR = 1.34 per SD increase, 95% CI 1.25–1.44, *p *= 1.48 × 10^−16^), CRPMort (*β* = 0.19, HR = 1.21, 95% CI 1.14–1.29, *p* = 9.58 × 10^−10^), and B2MMort (*β* = 0.078, HR = 1.08, 95% CI 1.03–1.14, *p *= 0.0028) were all significantly associated with all-cause mortality (Fig. 3b, Table S5).

We further examined associations with cause-specific mortality. GrimAge2Mort was significantly associated with mortality from heart diseases (*β* = 0.35, HR = 1.43, 95% CI 1.24–1.64, FDR = 2.44 × 10^−6^), malignant neoplasms (*β *= 0.32, HR = 1.38, 95% CI 1.18–1.60, FDR = 9.37 × 10^−5^), chronic lower respiratory diseases (*β* = 0.39, HR = 1.48, 95% CI 1.09–2.00, FDR = 0.019), diabetes (*β* = 0.48, HR = 1.62, 95% CI 1.18–2.24, FDR = 0.0059), influenza/pneumonia (*β* = 0.74, HR = 2.10, 95% CI 1.31–3.35, FDR = 0.0042), and other causes (*β* = 0.38, HR = 1.47, 95% CI 1.28–1.68, FDR = 1.66 × 10^−7^). CRPMort showed similar but generally weaker associations across these mortality categories, while B2MMort was specifically associated with heart disease mortality (*β* = 0.16, HR = 1.18, 95% CI 1.07–1.29, FDR = 0.0053) (Fig. 3c, Table S6).

To assess the predictive power of these DNAm features for mortality risk, we developed survival random forest models. The Harrell’s C-index values for all-cause mortality prediction were 0.69 for GrimAge2Mort, 0.689 for CRPMort, and 0.678 for B2MMort. A combined model incorporating all three features yielded a C-index of 0.691. Furthermore, time-dependent area under the receiver operating characteristic curves at 10-year survival revealed strong predictive performance: GrimAge2Mort (AUC = 0.73), CRPMort (AUC = 0.73), B2MMort (AUC = 0.72), and the combined model (AUC = 0.73), suggesting that these markers provide robust and overlapping mortality risk information (Fig. 3d).

### Dietary iron intake is directly associated with reduced mortality Risk

**Fig. 4:**
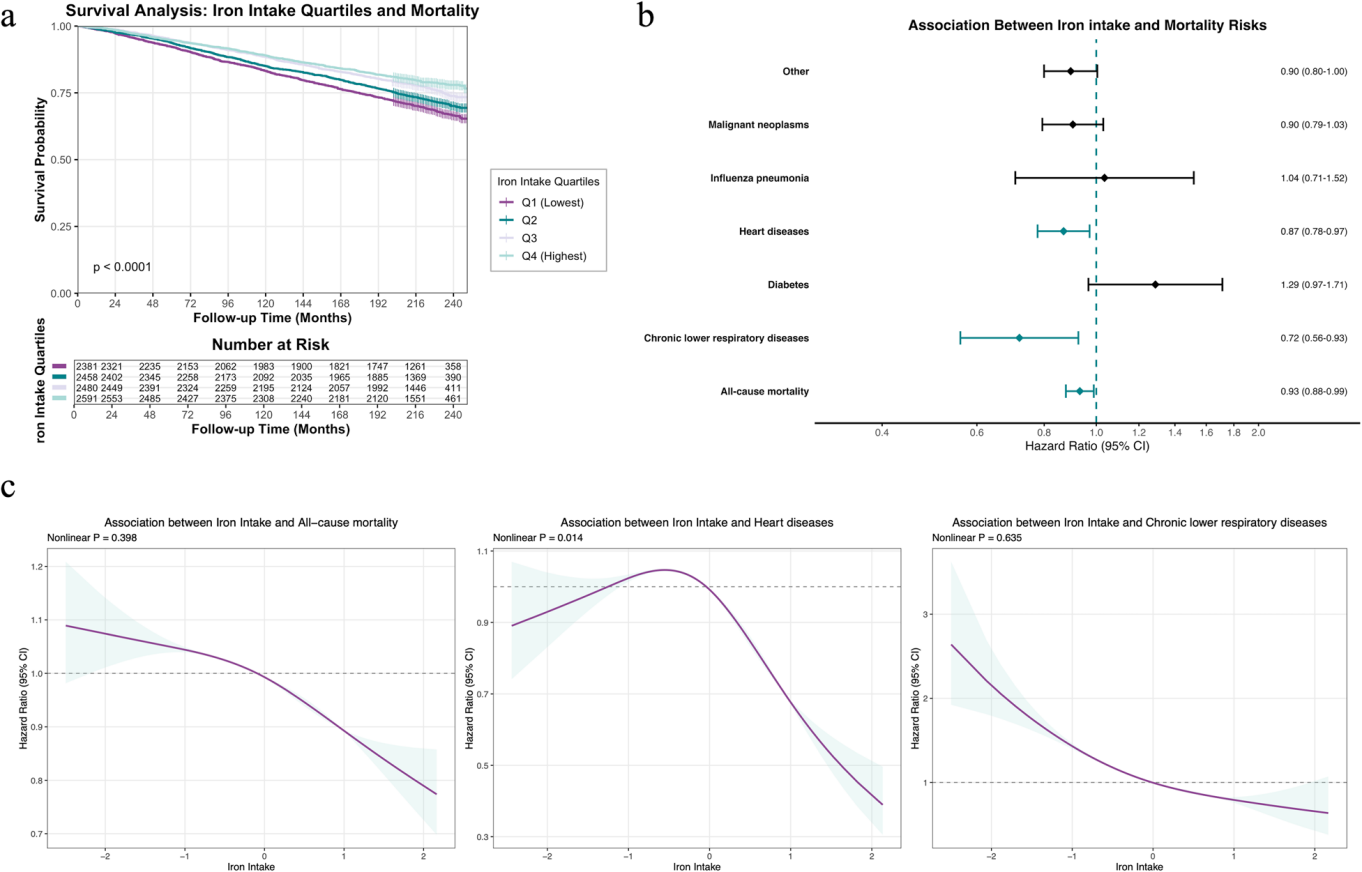
Relationship between dietary iron intake and mortality outcomes. **a,** Kaplan–Meier survival curves stratified by dietary iron intake quartiles, showing dose-dependent improvements in survival with increasing iron intake. **b,** Forest plot of hazard ratios (per standard deviation increase in iron intake) for all-cause and cause-specific mortality from fully adjusted Cox proportional hazards models. Green indicates statistically significant associations (*p* < 0.05). **c,** Restricted cubic spline models demonstrating nonlinear relationships between dietary iron intake and mortality risk, with reference set at median intake. Shaded areas represent 95% confidence intervals

To examine the direct relationship between iron intake and DNAm-responsive mortality outcomes, we generated Kaplan–Meier survival curves stratified by iron intake quartiles. Participants with higher iron intake consistently showed better survival across the follow-up period, with a clear dose–response relationship (Q4 > Q3 > Q2 > Q1) (Fig. 4a).

Cox regression analysis, adjusting for all covariates, revealed that higher iron intake (as a continuous variable) was significantly associated with reduced risk of all-cause mortality (HR = 0.93 per SD increase, 95% CI 0.87–0.99, *p* = 0.021), heart disease mortality (HR = 0.87, 95% CI 0.78–0.97, *p* = 0.014), and chronic lower respiratory disease mortality (HR = 0.72, 95% CI 0.56–0.93, *p* = 0.011) (Fig. 4b, Table S7).

When comparing iron intake quartiles (with Q1 as reference), we observed consistent protective effects for higher quartiles. Specifically, participants in Q4 showed significantly reduced risk of all-cause mortality (HR = 0.77, 95% CI 0.65–0.92, *p* = 0.002) and heart disease mortality (HR = 0.55, 95% CI 0.39–0.78, *p* < 0.001). Those in Q3 demonstrated reduced risk of all-cause mortality (HR = 0.83, 95% CI 0.71–0.98, *p* = 0.024), and chronic lower respiratory disease mortality (HR = 0.41, 95% CI 0.17–0.96, *p* = 0.04) (Table S8).

To explore potential nonlinear relationships, we constructed restricted cubic spline models for the significant mortality outcomes. These models confirmed predominantly negative associations between iron intake and mortality risk, with dose-dependent reductions in hazard ratios for all-cause mortality, heart disease mortality, and chronic lower respiratory disease mortality. While heart disease mortality showed a statistically significant nonlinear component (*p* < 0.05 for nonlinearity), the pattern still demonstrated reduced risk at higher iron intake levels, with nonlinearity primarily evident at low intake ranges (Fig. 4c).

### Iron intake modulates mortality risk through epigenetic mechanisms

**Fig. 5:**
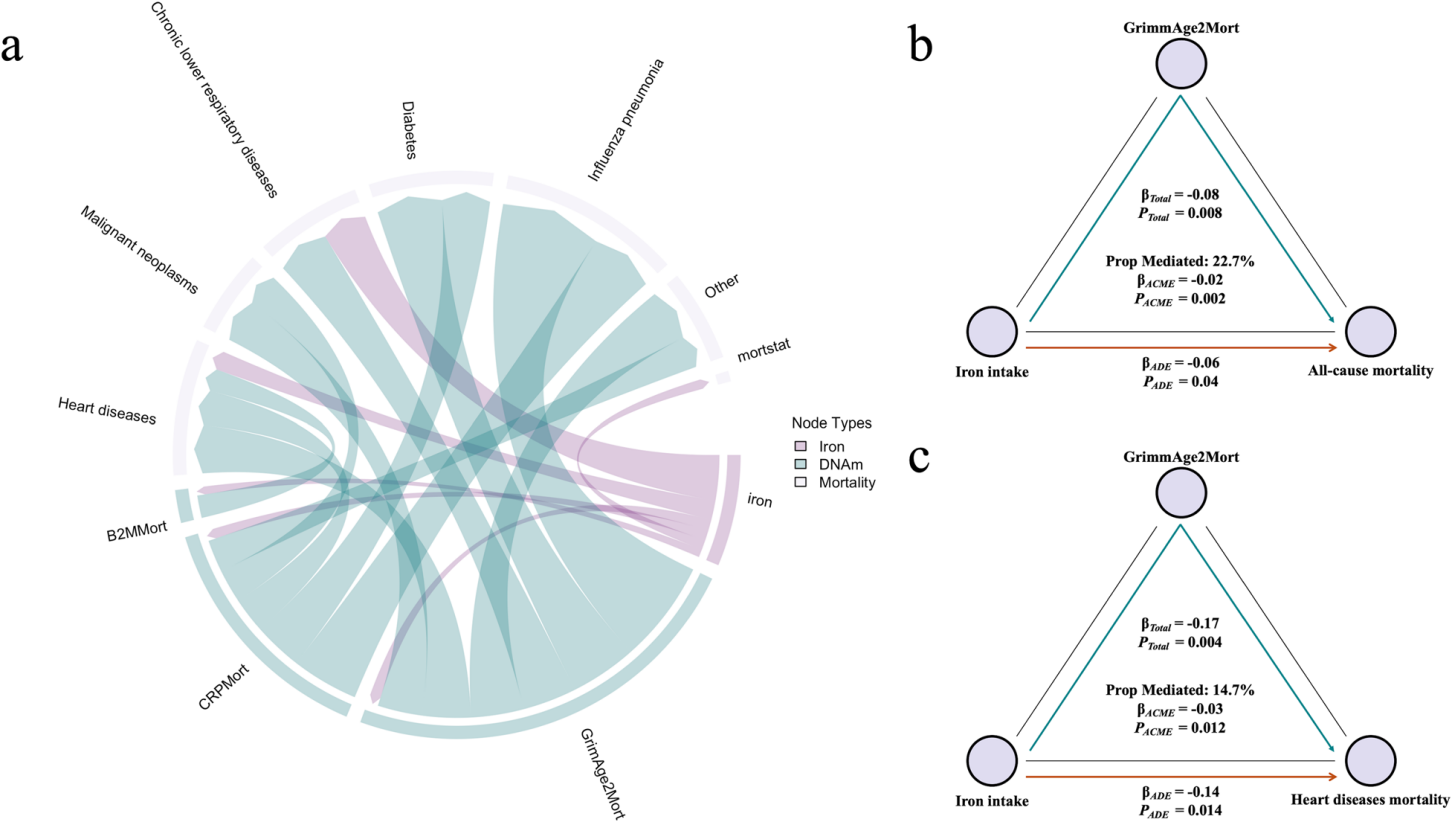
Epigenetic mediation of dietary iron’s effects on mortality risk. **a,** Chord diagram illustrating interconnections between dietary iron intake, DNA methylation features, and mortality outcomes. Width of connections represents strength of associations. **b,** Mediation analysis quantifying the proportion of iron intake’s effect on all-cause mortality mediated through GrimAge2Mort (25.8% mediated, *p* = 0.004). **c,** Mediation analysis for heart disease mortality, showing significant mediation through GrimAge2Mort (17.3% mediated, *p *< 0.001). ACME, average causal mediation effect; ADE, average direct effect

To integrate our findings on iron intake, DNA methylation, and mortality, we mapped potential mechanistic pathways using chord diagrams (Fig. 5a), representing the identified significant associations, showing the flow and strength of connections from dietary iron intake to specific DNA methylation features, and then from these features to various mortality outcomes. This analysis revealed multiple potential pathways connecting iron intake to reduced mortality risk via alterations in DNAm features, particularly through GrimAge2Mort, B2MMort, and CRPMort.

We formally tested these pathways using mediation analysis, adjusting for all covariates (as shown in Table S9). For all-cause mortality, GrimAge2Mort mediated 22.7% of the total effect of iron intake (ACME = −0.02, *p* = 0.002; total effect = −0.08, *p* = 0.008) (Fig. 5b) while CRPMort mediated 12.9% (ACME = −0.01, *p* = 0.008; total effect = −0.09, *p* = 0.006). For heart disease mortality, GrimAge2Mort mediated 14.7% of iron’s protective effect (ACME = −0.03, *p *= 0.012; total effect = −0.17, *p* = 0.004) (Fig. 5c), and CRPMort mediated 8.4% (ACME = −0.02, *p* = 0.034; total effect = −0.17, *p* = 0.002). B2MMort showed smaller mediation effects across all mortality outcomes, with a mediation proportion of 5.7% for all-cause mortality (ACME = −0.00, *p* = 0.046; total effect = −0.08, *p* = 0.018).

These findings suggest that iron’s beneficial effects on mortality risk are partially mediated through favorable alterations in DNA methylation profiles, particularly GrimAge2Mort and CRPMort, with the strongest mediation observed for all-cause and heart disease mortality.

### Subgroup analysis for baseline serum iron levels and cardiovascular disease status

To evaluate whether baseline iron status modifies the observed relationships, we first conducted interaction analyses stratified by baseline serum iron levels (quartiles). No significant interactions were detected between serum iron quartiles and iron intake-DNAm associations, DNAm-mortality relationships, or direct iron-mortality effects (all interaction *p* > 0.05; Tables S10-S12).

Additionally, given the prominent mediation effect observed for heart disease mortality (16.3%) and its status as the leading cause of death, we specifically tested interactions with baseline cardiovascular disease status (hypertension, heart disease, or stroke history). Similarly, no significant effect modification was observed for any iron-DNAm-mortality pathway (all interaction *p *> 0.05; Tables S13-S15).

These findings collectively indicate that the protective associations between dietary iron intake, epigenetic remodeling, and mortality risk remain consistent across the full spectrum of baseline iron status and cardiovascular health profiles in older adults. This suggests broad applicability of iron’s epigenetic benefits regardless of pre-existing metabolic conditions.

## Discussion

Our study systematically establishes, for the first time, the association between dietary iron intake and epigenetic aging markers, as well as the mediating role of these markers in the iron-mortality relationship by utilizing the NHANES cohort and long-term mortality follow-up data. Our findings demonstrate that higher dietary iron intake is linked to more favorable epigenetic profiles, particularly marked by significant reductions in mortality-associated DNA methylation markers, including GrimAge2Mort, CRPMort, and B2MMort. These iron-responsive epigenetic signatures not only robustly predict all-cause and cause-specific mortality but also mediate the protective effects of iron intake on mortality risk, with mediation proportions reaching 25.8% for all-cause mortality and 17.3% for cardiovascular disease mortality. Importantly, these associations remained consistent across varying baseline serum iron levels and cardiovascular disease statuses, underscoring that the epigenetic health benefits of iron intake extend broadly to diverse aging populations.

Our observation that dietary iron relates to reduced methylation-based mortality predictors aligns with emerging mechanistic frameworks. GrimAge2Mort, our strongest iron-responsive marker, represents a composite epigenetic predictor of physiological deterioration integrating multiple aging processes [27, 28]. The protective association between iron and GrimAge2Mort likely reflects iron’s fundamental role in cellular metabolic pathways. Specifically, iron serves as an essential cofactor for TET enzymes that catalyze active DNA demethylation through 5-methylcytosine oxidation—a process critical for maintaining appropriate methylation landscapes [29–31]. Experimental evidence suggests that iron deficiency impairs TET activity, potentially accelerating age-related dysregulation of methylation patterns [32, 33]. Conversely, adequate iron availability may preserve methylation homeostasis by supporting optimal TET function [34].

The iron-associated reduction in CRPMort—an epigenetic surrogate of systemic inflammation [27, 28]—further suggests that appropriate iron intake may attenuate inflammaging, a primary driver of age-related pathologies. While excessive iron can potentially promote inflammation through Fenton chemistry and oxidative stress [35, 36], our findings suggest that within the observed dietary range, iron’s anti-inflammatory benefits predominate, possibly through optimal heme synthesis and oxygen transport that prevents tissue hypoxia-induced inflammation [37, 38]. The absence of adverse methylation signatures even at higher intake quartiles suggests that typical dietary iron levels rarely reach the pro-oxidant threshold in generally healthy older adults.

The consistent inverse associations between iron intake and mortality outcomes, particularly for cardiovascular and respiratory causes, highlight iron’s potential as a modifiable determinant of longevity. The observed dose–response relationship for all-cause mortality (7% reduction per standard deviation increase in iron intake) suggests clinical relevance at population scale. Our findings contextualize seemingly contradictory prior research: while iron deficiency and overload both associate with increased mortality [14, 15], we demonstrate that within typical dietary ranges, higher iron intake correlates with reduced mortality risk through epigenetic pathways distinct from anemia or hemochromatosis mechanisms.

The pronounced cardiovascular protection observed (13% risk reduction per SD increase in iron intake) merits particular attention given heart disease’s position as the leading mortality cause. This cardioprotective effect may operate through multiple mechanisms beyond traditional iron deficiency pathways. Adequate iron availability supports mitochondrial function within cardiomyocytes, facilitates nitric oxide signaling for vascular homeostasis, and enables efficient oxygen transport [39–41]—functions potentially reflected in the favorable methylation profiles we observed. The substantial mediation effect (17.3%) through GrimAge2Mort for cardiovascular mortality suggests epigenetic remodeling represents a key molecular pathway translating nutritional iron exposure to cardiovascular resilience.

Our mediation analyses provide the first population-level evidence that epigenetic recalibration constitutes a substantial mechanism linking nutritional iron to mortality outcomes. The identification of specific methylation features mediating iron’s protective effects advances the field beyond correlation toward causal pathways. GrimAge2Mort’s stronger mediation effect compared to CRPMort and B2MMort suggests that iron influences mortality risk predominantly through composite aging processes rather than singular inflammatory or cellular senescence pathways.

This partial mediation indicates iron likely operates through multiple complementary mechanisms beyond methylation alone. Unmediated pathways may include direct effects on oxygen transport efficiency, energy metabolism, immune function, and neurotransmitter synthesis—all processes where iron serves critical roles [42–44]. The synergy between these direct physiological effects and the epigenetic pathways we identified may explain the robust protective associations observed across diverse mortality causes.

These findings have immediate implications for nutritional recommendations in aging populations. Current dietary guidelines for older adults primarily address iron requirements for preventing deficiency anemia but largely overlook iron’s broader role in healthy aging and mortality risk. Our results suggest optimizing iron intake may represent an accessible, low-cost intervention to decelerate epigenetic aging and reduce mortality risk. Notably, our comprehensive subgroup analyses (Tables S9-S14) revealed that the protective associations between dietary iron intake, epigenetic remodeling, and mortality risk remained consistent across varying baseline serum iron levels and cardiovascular disease statuses (all interaction *p* > 0.05). This lack of significant interaction indicates that the beneficial effects of dietary iron are broadly applicable, suggesting that individuals across the spectrum of baseline iron status, including those with lower baseline levels who might benefit from restored physiological levels, or those with higher baseline levels, can derive similar epigenetic and survival advantages from optimizing their dietary iron intake. This broad applicability underscores the potential for dietary iron as a universal strategy to promote healthy aging, regardless of pre-existing iron status or common chronic conditions.

The absence of effect modification by baseline iron status or cardiovascular disease is particularly encouraging, suggesting dietary optimization may benefit diverse subpopulations regardless of pre-existing conditions. Implementation could involve both population-level approaches (food fortification programs targeting older adults) and personalized recommendations using epigenetic aging markers to identify individuals most likely to benefit from iron supplementation or dietary modification.

Our study offers several notable strengths. First, we leveraged nationally representative samples with mortality follow-up spanning 20 years, providing robust epidemiological evidence for the iron-epigenetics-mortality relationship. Second, we employed a comprehensive approach to assess epigenetic aging by integrating 30 established DNA methylation markers and selecting 17 relatively independent features through rigorous dimensionality reduction techniques, ensuring result robustness. Third, we utilized advanced statistical methods to evaluate nonlinear associations and mediation effects, revealing potential mechanisms through which iron influences mortality. Fourth, our analyses accounted for multiple potential confounders including demographic characteristics, socioeconomic factors, lifestyle habits, and baseline health conditions, minimizing bias. Finally, we validated the consistency and robustness of our findings through stratified analyses demonstrating that the epigenetic benefits of iron intake remain stable across different baseline iron statuses and cardiovascular health conditions.

Despite providing compelling evidence for associations between iron intake, epigenetic aging, and mortality, several limitations warrant consideration. First, as an observational study, we cannot establish causality, although mediation analyses provide mechanistic clues. Future randomized controlled trials testing iron interventions on epigenetic aging will be crucial for establishing causal relationships. Second, dietary iron intake based on 24-h dietary recalls may not fully capture long-term exposure patterns. Multi-timepoint dietary assessments and biomarker measurements would provide a more comprehensive picture of iron status. Third, DNA methylation data derived only from peripheral blood may not reflect tissue-specific epigenetic changes. Tissue-specific epigenomic analyses would further elucidate iron’s role across different organ systems. Fourth, despite controlling for multiple confounders, we cannot exclude residual confounding, particularly from unmeasured nutritional factors or dietary patterns. Finally, the exact details of molecular mechanisms remain incompletely elucidated and require functional experimental validation.

## Conclusions

In conclusion, this study establishes dietary iron intake as a modifiable determinant of epigenetic aging and mortality risk, with epigenetic recalibration mediating a substantial proportion of iron’s protective effects. These findings challenge simplistic views of iron’s health impacts and highlight the necessity of reconsidering nutritional guidelines for aging populations. By identifying specific epigenetic pathways linking iron to mortality outcomes, our research provides not only biomarkers for risk stratification but also intervention targets for extending healthy longevity. These results position iron optimization as a promising and accessible strategy within the growing arsenal of interventions aimed at compressing morbidity and preserving function.

## Supplementary Information

Additional file 1.

Additional file 2.

## Data Availability

No datasets were generated or analyzed during the current study.

## Abbreviations:

ACME: Average causal mediation effect
ADE: Average direct effect
AUC: Area under the receiver operating characteristic curve
BMI: Body mass index
CI: Confidence interval
DNAm: DNA methylation
EDF: Estimated degrees of freedom
FDR: False discovery rate
GAMs: Generalized additive models
HR: Hazard ratio
NHANES: National Health and Nutrition Examination Survey
NDI: National Death Index
PCA: Principal component analysis
PIR: Poverty-income ratio
PH: Proportional hazards
Q: Quartile
RCS: Restricted cubic spline
SD: Standard deviation
TET: Ten-eleven translocation
