# Supplementary material for: Dietary iron attenuates epigenetic aging through DNA methylation remodeling and extends survival in older adults
Source: Clin Epigenetics. 2025 Oct 29;17:181. doi: 10.1186/s13148-025-01986-x (PMC12573944; doi:10.1186/s13148-025-01986-x)
Supplement: Supplementary file 1 — Additional file 1. [file 13148_2025_1986_MOESM1_ESM.docx]

**Figure S1. Distributions of dietary iron intake​.**

**a.** Raw intake distribution. Empirical density of daily iron intake (mean ± SD = 14.02 ± 8.43 mg/day). **b.** Transformed intake distribution. Normalized density after log transformation and z-score standardization.


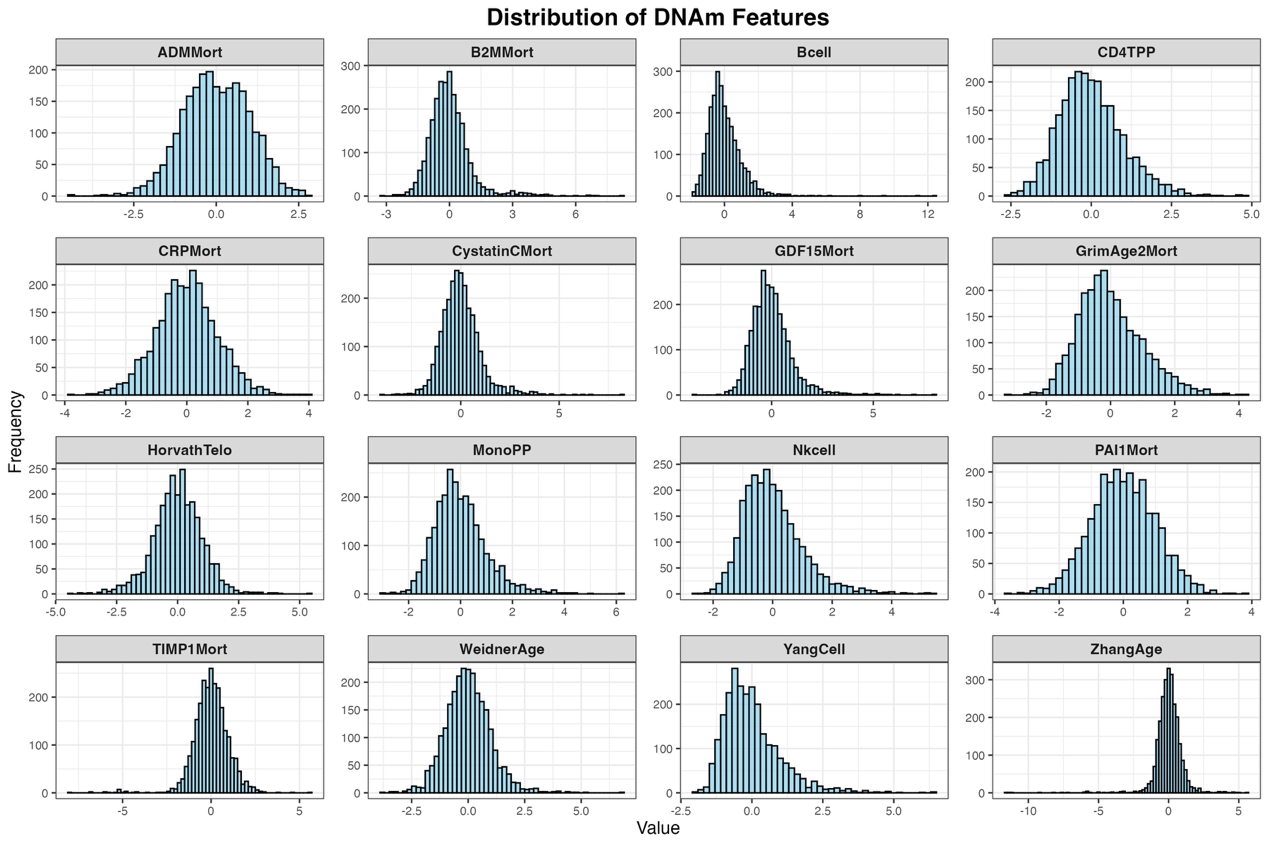


**Figure S2 Distributions of DNA methylation biomarkers​.**

Density plots of z-scored residuals after regressing each biomarker on chronological age.

**
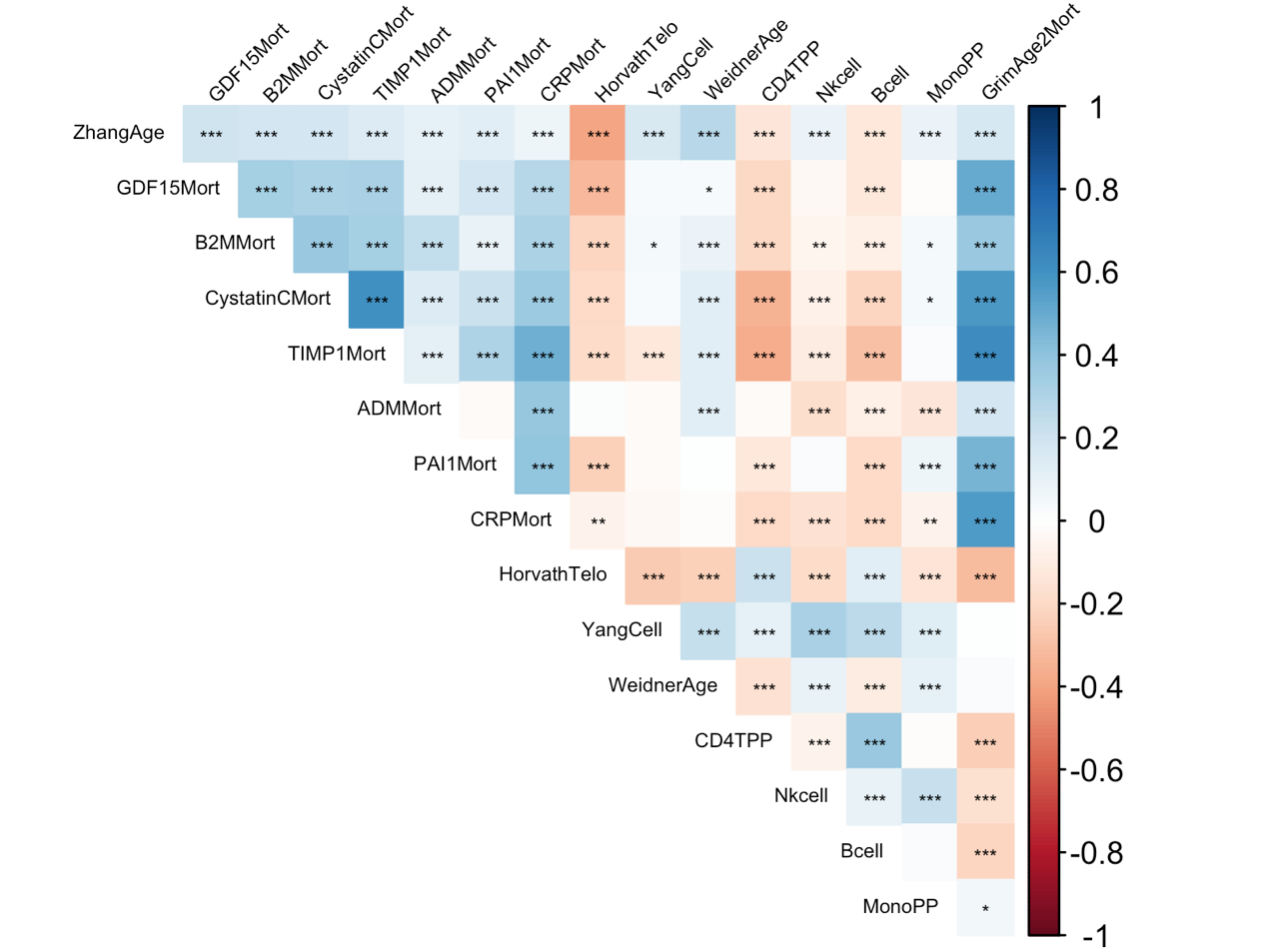
**

**Figure S3 Correlation matrix of 17 DNA methylation biomarkers.**

Spearman’s rank correlations (ρ) between age-residualized DNAm z-scores. Features were selected via clustering (70% dissimilarity threshold). ​*p<0.001, ​p<0.01, *p<0.05.

**
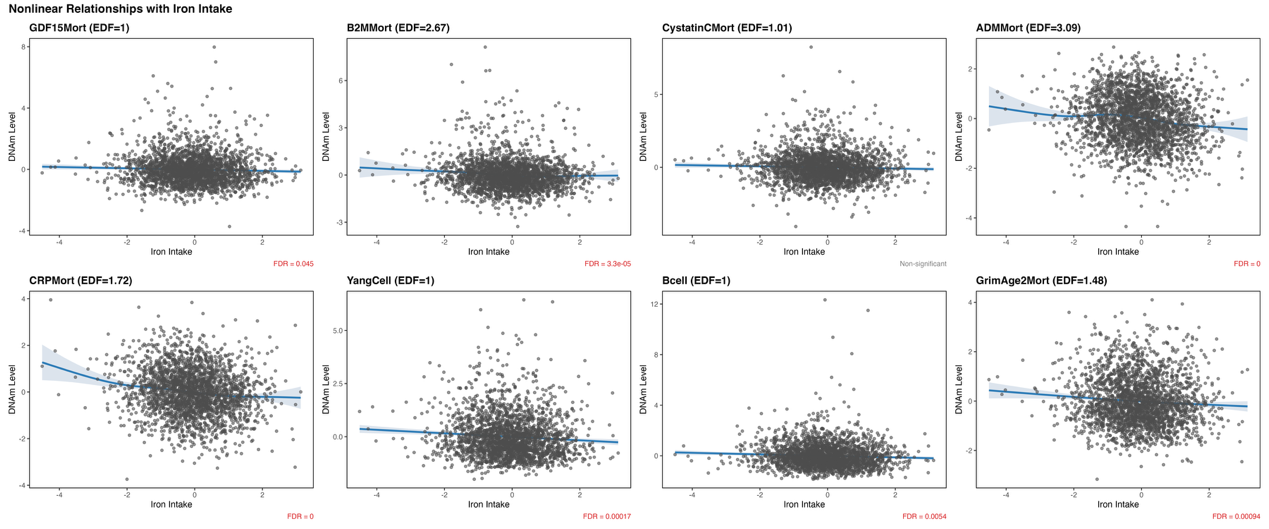
**

**Figure S4 Non-linear associations between iron intake and DNAm features.**

Generalized additive models (GAM) of iron z-scores versus DNAm features. Solid lines: smoothed fits; shaded regions: 95% CIs. Annotated EDF (effective degrees of freedom) indicates non-linearity magnitude.
